# Optimized 3D co-registration of ultra-low-field and high-field magnetic resonance images

**DOI:** 10.1371/journal.pone.0193890

**Published:** 2018-03-06

**Authors:** Roberto Guidotti, Raffaele Sinibaldi, Cinzia De Luca, Allegra Conti, Risto J. Ilmoniemi, Koos C. J. Zevenhoven, Per E. Magnelind, Vittorio Pizzella, Cosimo Del Gratta, Gian Luca Romani, Stefania Della Penna

**Affiliations:** 1 Department of Neuroscience, Imaging and Clinical Science, Chieti, Italy; 2 Institute for Advanced Biomedical Technologies, University G. D’Annunzio of Chieti and Pescara, Chieti, Italy; 3 Department of Neuroscience and Biomedical Engineering, Aalto University School of Science, FI, Aalto, Finland; 4 Applied Modern Physics Group, Physics Division MS-D454, Los Alamos National Laboratory, Los Alamos, NM, United States of America; 5 Consiglio Nazionale delle Ricerche, Istituto SPIN, UOS L’Aquila, Sede di lavoro CNR-SPIN c/o Università di Chieti-Pescara “G. D’Annunzio”, Chieti, Italy; University of Chicago, UNITED STATES

## Abstract

The prototypes of ultra-low-field (ULF) MRI scanners developed in recent years represent new, innovative, cost-effective and safer systems, which are suitable to be integrated in multi-modal (Magnetoencephalography and MRI) devices. Integrated ULF-MRI and MEG scanners could represent an ideal solution to obtain functional (MEG) and anatomical (ULF MRI) information in the same environment, without errors that may limit source reconstruction accuracy. However, the low resolution and signal-to-noise ratio (SNR) of ULF images, as well as their limited coverage, do not generally allow for the construction of an accurate individual volume conductor model suitable for MEG localization. Thus, for practical usage, a high-field (HF) MRI image is also acquired, and the HF-MRI images are co-registered to the ULF-MRI ones. We address here this issue through an optimized pipeline (SWIM—Sliding WIndow grouping supporting Mutual information). The co-registration is performed by an affine transformation, the parameters of which are estimated using Normalized Mutual Information as the cost function, and Adaptive Simulated Annealing as the minimization algorithm. The sub-voxel resolution of the ULF images is handled by a sliding-window approach applying multiple grouping strategies to down-sample HF MRI to the ULF-MRI resolution. The pipeline has been tested on phantom and real data from different ULF-MRI devices, and comparison with well-known toolboxes for fMRI analysis has been performed. Our pipeline always outperformed the fMRI toolboxes (FSL and SPM). The HF–ULF MRI co-registration obtained by means of our pipeline could lead to an effective integration of ULF MRI with MEG, with the aim of improving localization accuracy, but also to help exploit ULF MRI in tumor imaging.

## 1. Introduction

In the last 15 years, new instrumental apparatuses have been developed to perform Magnetic Resonance Imaging (MRI) at low fields (LF MRI, *B* < 10 mT), in contrast to the general tendency to increase the magnetic field strength for higher spatial resolution (see [1,2] for reviews on actual LF NMR/MRI instruments and applications). These devices operate at field levels ranging from ~1 μT (Ultra Low Field, ULF [3,4]) to ~10 mT (Very Low Field, VLF [3–6]).

The advantages of LF MRI include: i) safer operation for specific patient populations (children, pregnant women, patients with metallic prostheses or electronic implants), due to the lower static field; ii) detection in the presence of metals, thanks to the reduced sensitivity to magnetic susceptibility; iii) lower cost compared to HF devices; iv) possibility of integration with other instruments such as Magnetoencephalography (MEG) systems [7,8]; v) possibility of directly imaging neural currents in cerebral regions responding to a stimulus [9]; and vi) enhanced contrast of the relaxation time *T*_1_ at low static magnetic fields. In particular, ULF MRI has been proven to be able to differentiate healthy and cancerous tissues on the basis of the *T*_1_ relaxation time without the need of contrast agents [10,11].

The integration of LF-MRI and MEG devices and the subsequent possibility to assess the anatomical brain structure and to record MEG activity with the same instrument [7,12] would remove the contribution of the MEG and MRI co-registration error considerably improving MEG spatial accuracy, which is often worse than 5 mm. Drawbacks of the existing LF-MRI prototypes are the lower spatial resolution and lower signal-to-noise ratio (SNR) than in high-field scanners (HF MRI). Additionally, due to the use of pulsed pre-polarization techniques to increase the SNR [13–16], acquisition times are considerably longer than in HF MRI, so that only part of the subject’s head is usually scanned. As a consequence, today’s ULF images of the brain cannot be directly used to construct the volume conductor model for MEG. A possible solution to this problem is to acquire a separate set of HF-MR images covering the whole head, for reference, and then to co-register these images to the ULF-MR ones. To effectively improve the MEG localization error, it is of paramount importance that the co-registration between the HF-MRI and the original LF-MRI be performed as accurately as possible.

Well-assessed co-registration procedures are already available for specific types of biomedical images, such as multimodal images acquired with different devices, e.g., MRI and CT or MRI and PET, the outputs of which are co-registered in the same standard space [17,18]. Among the existing procedures, the best candidates able to co-register LF and HF MRIs are those used to co-register functional and anatomical MRIs produced by the same scanner and implemented in fMRI processing software (e.g., SPM, FSL and many others; [19,20]).

To this end, Mutual Information (MI) based algorithms [21–23] are widely used to find the best co-registration transformation. In particular, MI was demonstrated to be optimal for multimodal data analysis where images recorded from different devices or with different contrasts, such as fMRI and MRI, must be aligned in a common space, whereas many other mathematical cost functions can be misleading when handling images with different SNR and sensitivity [24]. Of note, this approach was tested for co-registering fMRI and MRI images with high SNR and contrast on the whole brain volume. On the contrary, if the image SNR is low, such as in current ULF MRI, then the cost function will have more local minima where the minimization process could be trapped [18], thus disturbing the co-registration process.

To the best of our knowledge, there is no efficient tool for the co-registration of low-resolution, low-contrast images with high-contrast, high-resolution 3D images. Ultrasound images used for multimodal co-registration have also low SNR, but they have a high spatial resolution [25,26]. Additionally, this tool should co-register the whole volume imaged with HF systems to only a portion of this volume recorded by ULF MRI.

We present here a new pipeline (SWIM—Sliding WIndow grouping supporting Mutual information) specifically designed to co-register HF MRIs to LF MRIs, with a low SNR, low contrast, low resolution, and only partial coverage of the imaging volume. This pipeline is based on Normalized Mutual Information (NMI) [17], supported by an accurate sub-voxel sampling procedure based on a sliding window. The results obtained on three different datasets were analyzed to validate this method. Specifically, we carried out a first test on datasets of a phantom with a known geometry. We then performed two tests on *in-vivo* human brain data recorded at Los Alamos National Laboratory [12], and at AALTO University [7]. Our results were then compared with those from standard software for the co-registration of fMRI to anatomical images.

## 2. Materials and methods

**Ethics statement:** The MRI experiments performed at AALTO University were approved by the Ethics Committee of the Hospital District of Helsinki and Uusimaa. The human subject experiments, acquired at Los Alamos, were approved by the Los Alamos Institutional Review Board.

### 2.1 SWIM Co-registration pipeline

#### 2.1.1 Mutual information based registration

The goal of the image co-registration is to find the set of parameters *t*, defining the transformation *T* that brings an image *A* into the best possible spatial correspondence with a second image *B*.

$$\arg\underset{t}{\min}\left(B-TA\right)$$

The affine transformation *T* can be composed by a product of multiple independent transformations; here, *T* consists of a rigid transformation defined by 6 parameters representing 3 translations and 3 rotations around the *x*, *y*, *z* axes of the reference system of image *A*, and a scaling transformation to match for possible anisotropic distortions across images with 3 parameters, one for each spatial dimension. From now on, we will name as *rigid* the transformation with 6 parameters and as *rigid+scaling* the transformation with both rigid and scaling parameters with a total of 9 parameters.

According to information theory and literature on image analysis [27], MI is a suitable cost function to find the best transformation T*. Specifically, the MI criterion states that T* maximizes the MI between *A* and *B*.

For two images *A* and *B*, MI is defined as follows:

MI(A,B)=H(B)+H(A)−H(A,B)=H(A)−H(A|B)=H(B)−H(B|A) (1)

Where *H(X)* is the Shannon entropy of image *X*, *H*(*A*|*B*) is the conditional entropy of *A* given *B* and *H*(*A*, *B*) is the joint entropy, defined as in the following:

$$H(A)=-\sum_{a}p_{A}(a)\;\log p_{A}(a)$$ (2)

$$H(A,B)=-\sum_{a,b}p_{AB}(a,b)\;\log p_{AB}(a,b)$$ (3)

$$H(A|B)=-\sum_{a,b}p_{AB}(a,b)\;\log p_{A|B}(a|b)$$ (4)

Here, *p*_*A*_(*a*) and *p*_*B*_(*b*) are the marginal probability distributions of images *A* and *B*, obtained as the frequency of a grey voxel intensity *y* in an image *Y*. The joint probability distribution *p*_*AB*_(*a*, *b*) is obtained from the joint histogram of *A* and *B*, where each {*i*, *j*} entry represents the frequency of a voxel intensity *i* in the first image (*A*) and voxel intensity *j* in the second image (*B*) at the same coordinate.

The probability distribution *p*_*AB*_(*a*, *b*) depends on the alignment between the two images, and thus on the transformation *T* applied to image *A*.

SWIM uses a modified MI, the Normalized Mutual Information (*NMI*) [17], which has been demonstrated to be less sensitive than MI (Eq 1) to the size of the overlapping part of the two 3D structures; it is better suited for our requirements (see previous subsection). *NMI* is defined as:

$$NMI(A,B)=\frac{H(A)+H(B)}{H(A,B)}$$ (5)

In addition, SWIM uses a reverse mapping procedure [28] and a trilinear interpolation [29] to prevent the generic transformation *T* from creating empty voxels in the transformed image, altering the NMI value [30]. These empty voxels could be caused by the discrete sampling of the image space, implying that after a generic transformation *T*, the new position of each voxel may not coincide with a point on the grid described by the coordinate set. Moreover, numerical truncation may lead to two or more voxels being transformed in the same one after direct transformation.

#### 2.1.2 Minimization and grouping procedures

We used Adaptive Simulated Annealing (ASA) [31] to find the parameters of the transformation T* that minimize the reciprocal 1/*NMI*. *NMI*^*–1*^ is not a convex function of the set of parameters *t*, and although it has been shown that there is no optimization method that is superior to others [32], ASA is a robust method less prone to be trapped in local minima or funnels [31] that characterize the space of co-registration parameters for noisy and low-contrast images. We ran the ASA algorithm 10 times, each with a different starting configuration, using 500 runs for each configuration.

There is an additional issue we had to face during minimization of 1/*NMI*. Since *NMI*-based co-registration must be applied to pairs of images with the same spatial resolution, we down-sampled the HF images to the ULF image resolution. To this aim, we applied a grouping procedure, namely the sliding window approach, as described in the following: In the HF images, once a voxel was selected as the origin of the HF grid, we averaged in each direction a number of voxels equal to the ratio between the voxel size of the low- and high-resolution images. The total number of voxels to be averaged in the 3D grouping window $\left(N_{3D}^{av}\right)$ is equal to the product of the ratio between the voxel size of the low- and high-resolution images in three dimensions:

$$N_{3D}^{av}=\prod_{d=x,y,z}\left\lceil \frac{size(v_{d}^{LR})}{size(v_{d}^{HR})}\right\rceil$$ (6)

In Eq (6), the *size* function gives the resolution of voxel *v* in the *d* dimension for low-resolution (LR) and high-resolution (HR) images. The size of the 3D window described in Eq (6) is fixed. However, the co-registration accuracy depends on the position of the 3D window. Before the ASA minimization procedure, the 3D window can be slid onto the high-resolution image: this implies that the voxels constituting the grouping window depend on three translation parameters. The number of trials for the selection of the best position of the grouping window is equal to the number of voxels included in the window.

**Fig 1:**
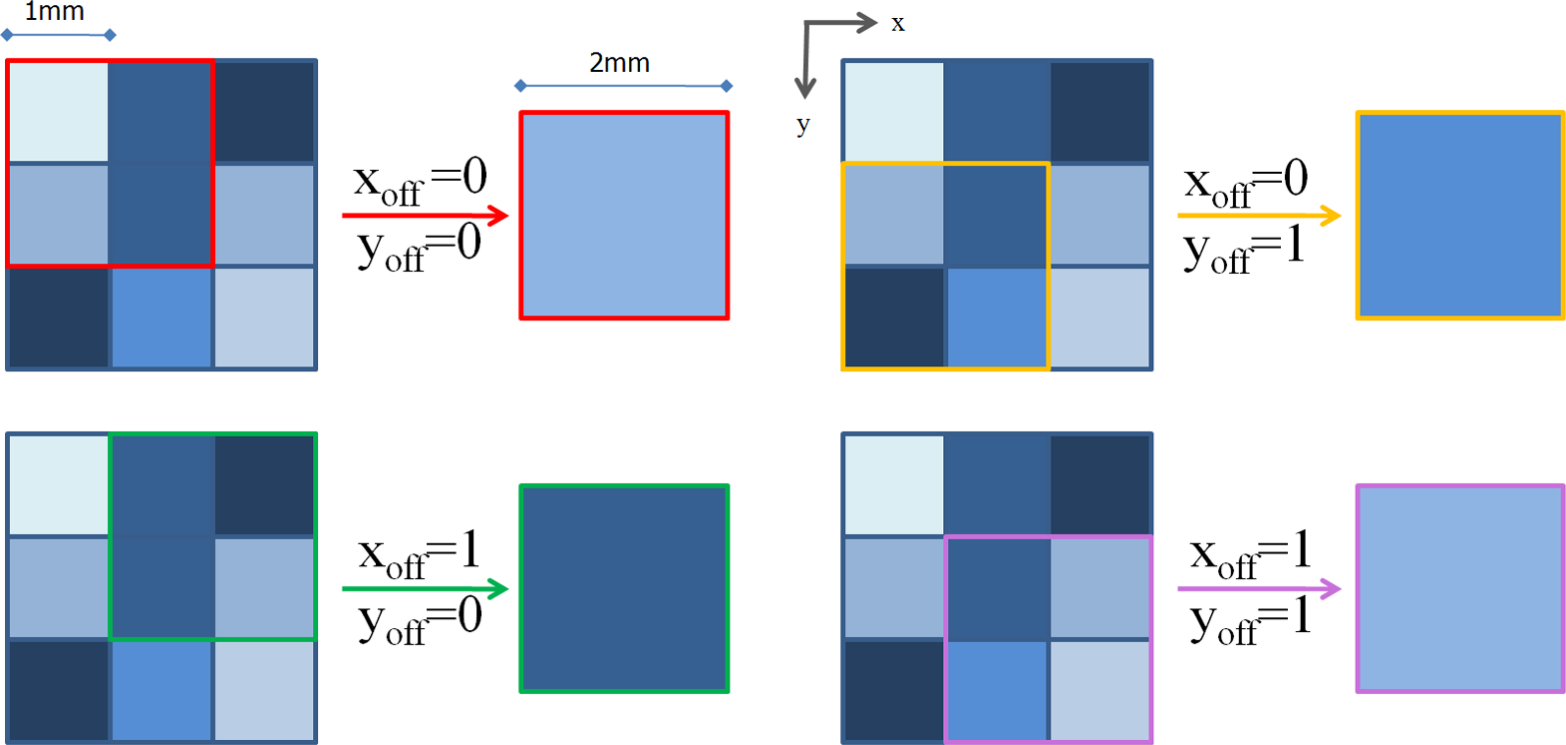
Schematic drawing illustrating the grouping procedure. The figure shows an example of the grouping procedure to down-sample a 2D image with a voxel size 1×1 mm^2^ into another one with 2x2mm^2^ resolution. We have four possible groupings, shown here in different colors (red, green, orange and pink). Each grouping relates to a different offset out of the possible four. Notably, at each grouping window a different value is obtained when voxels are averaged, showing how this procedure modifies the information content of the image, particularly when the image to be down-sampled has a large number of edges.

This effect is illustrated in Fig 1 in a simple 2D case: an image with a voxel size equal to 1×1 mm^2^ is down-sampled to a resolution of 2×2 mm^2^. We have four possible 2D window positions (from Eq 6) to down-sample the image with voxel size 1×1 mm^2^. In the figure, each offset individuates a window with a different edge color and it is clear that different offsets produce different average values over the 2D window and thus different image contents. In general, a shift in one direction will modify the group of voxels participating at each 3D window, so that the number of voxels constituting the intersection between the shifted and 0-shift windows is equal to

$$\prod_{d=x,y,z}\left(\lceil \frac{size(v_{d}^{LR})}{size(v_{d}^{HR})}\right\rceil -{offset}_{d})$$ (7)

This number could be relatively small, thus suggesting how the position of the grouping sliding window influences the mutual information value and the whole co-registration process.

In summary, the SWIM pipeline explores all possible positions of the sliding window and selects the most informative tessellation for the image-registration algorithm. Eventually, a minimization run is performed for different tessellations, searching for the highest NMI value, which corresponds to the best transformation found by the algorithm. In this optimization step, only three translational parameters are involved and a minimization procedure based on the *Downhill Simplex Method* in multi-dimensions [33] is rather efficient and less time consuming than ASA. We used 500 runs for the optimization pipeline.

The output is the set of transformed high-resolution images to be superimposed with the low-resolution one, and the best NMI value obtained. For qualitative evaluation of the co-registration results, we computed the difference images between the two sets of aligned image stacks at low spatial resolution. This requires that a threshold is applied on both ULF and HF image stacks. This threshold is automatically selected using an adaptive procedure based on the image histogram [28,34].

All the algorithms were written in C and ran on a standard Linux desktop PC with a quad-core CPU and 4 GB of RAM.

Two different open-source software packages, commonly used in the neuroscience community, were used to compare and validate our approach: FSL [20] and SPM [35]. In this comparison, the NMI values were calculated using the output images of all the different software tools as input to SWIM, without any optimization, to be sure that the calculation of the NMI value was performed exactly with the same procedure and using the same volumes.

In addition to NMI values, we also calculated a group of image similarity indices to further evaluate the co-registration. These indices are commonly used in the image-processing literature [36,37]; their mathematical formulations are reported in Table 1.

**Table 1:** Mathematical formulation of similarity measures. We used these measures to evaluate the performances of SWIM. In the first column containing the index names, S stands for similarity measure while D stands for dissimilarity measure.

Jaccard (S)	$\frac{\sum_{i=1}^{n}min(a_{i},b_{i})}{\sum_{i=1}^{n}max(a_{i},b_{i})}$		
R_2_ score (S)	$1-\frac{\sum_{i=1}^{n}{\left(b_{i}-\bar{a}\right)}^{2}}{\sum_{i=1}^{n}{\left(a_{i}-\bar{a}\right)}^{2}}$		Where $\bar{a}=\frac{1}{n}\sum_{i}^{n}a_{i}$
Kendall tau (S)	$\frac{C-D}{\frac{n(n-1)}{2}}$	C is the number of concordant pairs while D is the number of discordant pairs. A pair [(a_i_, bi), (a_j_, bj)] is defined concordant if sign(a_i_-a_j_) = sign(b_i_-b_j_). They are said to be discordant, in other cases.	
Bray Curtis (D)	$\begin{array}{c} \frac{\sum_{i=1}^{n}{(a}_{i}-b_{i})}{\sum_{i=1}^{n}\left(a_{i}+b_{i}\right)} \end{array}$		
Mean Squared Error (D)	$\frac{1}{n}\sum_{i=1}^{n}{\left(a_{i}-b_{i}\right)}^{2}$		
Correlation distance (D)	$1-\frac{\sum_{i=1}^{n}\left(a_{i}-\bar{a})(b_{i}-\bar{b}\right)}{\sqrt{\sum_{i=1}^{n}(a_{i}-\bar{a})\sum_{i=1}^{n}\left(b_{i}-\overset{´}{\bar{b}}\right)}}$		

### 2.2 Datasets

#### 2.2.1 Phantom images acquired at 8.9 mT and at 3T

We first tested the SWIM pipeline on MR images of a phantom with a known 3D geometry, which is free from possible image blurring and motion artifacts. The phantom was a hollow cylinder with an asymmetrical hole filled with doped water (770 mg of CuSO_4_ in 1 dm^3^ of H_2_O, 1 ml Arquad, 0.15 ml H_2_SO_4_), and contained in a volume of 5×5×4 cm^3^. HF-MR images were recorded on a Philips 3T scanner using a knee coil and an Ultra Fast Gradient Echo sequence, with 1×1×1 mm^3^ resolution, 12×12×18 cm^3^ FOV, TR = 8.5 ms, TE = 3.9 ms, NEX = 3, and total acquisition time = 6 min.

VLF MRIs were recorded on a prototype operating at a static field of 8.9 mT inside a magnetically shielded room [6]. We used a Spin Echo (SE) sequence, cartesian sampling with 1×1×1 mm^3^ resolution, 6.4×6.4×6.4 cm^3^ FOV, TR = 500 ms, TE = 19 ms, NEX = 37 and total acquisition time of 8.5 min for each volume. To save time, only 32×32 phase encoding steps and zero filling were used. Note that in this first dataset, the whole phantom was imaged with the LF and HF devices.

#### 2.2.2 Brain images acquired at 46 μT and at 1.5 T

Our pipeline was applied to a second dataset consisting of images of a human brain acquired with HF- and ULF-MRI scanners at Los Alamos National Laboratory (LANL), Applied Modern Physics Group [12]. The HF dataset was acquired with a conventional 1.5 T scanner using an SE sequence with TE = 64 ms and TR = 9000 ms. The HF image size is 256×256×192 cubic voxels, with side length of 1 mm. The ULF-MRI brain dataset was acquired in a static field of 46 μT, using pre-polarizing pulses of 30 mT [12]. The voxel resolution of the ULF 3D acquisition was 6×3×3 mm^3^, NEX = 6. The detection coil array was placed above the right temporal lobe. The total number of voxels was 6 in the *x* direction, 45 in the *y* direction and 40 in the *z* direction. Note that, as observed above, only a portion of the left hemisphere was imaged with ULF MRI, and this portion had to be co-registered to the whole head volume acquired with the HF set-up.

#### 2.2.3 Brain images acquired at 50 μT and at 3 T

Finally, we tested SWIM on a dataset acquired with the ULF-MRI apparatus installed at Aalto University [7]. Images of the brain were acquired with a static field of 50 μT and a prepolarization field of 22 mT, using a 3D SE sequence with NEX = 8. The detection coils were placed above the occipital lobe. The resolution was 4×6×4 mm^3^ and an image matrix of 50×16×38 voxels was reconstructed. In this case as well, only a portion of the brain was imaged at ULF. The HF MRI of the brain was acquired with a Siemens 3T MRI scanner, using a Turbo SE with a TR = 4920 ms and TE = 121 ms. The HF images had a voxel resolution of 1×5×1 mm^3^, resulting in an image grid composed of 256×79×256 voxels.

## 3. Results

### 3.1 Phantom data recorded at 8.9 mT and 3T

**Fig 2:**
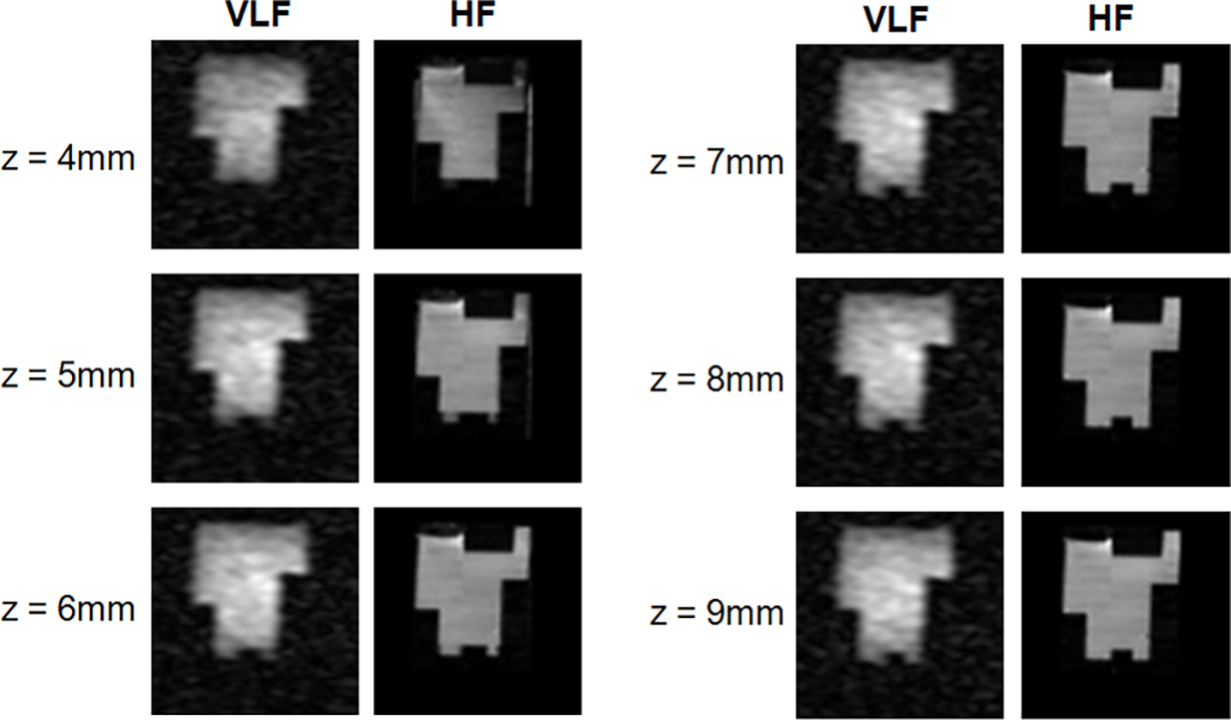
Co-registration of the phantom dataset. Images of the phantom dataset acquired at VLF (left panel) and at HF, after co-registration (right panel) at 6 different depths (4–9 mm).

In Fig 2, we show the result of the SWIM pipeline applied on the phantom images acquired at HF and VLF. Although in these images the signal is generated by the doped water in the phantom, they differ due to the following reasons: *i*) the respective upper parts of the images are different since the phantom was closed by a cap during the HF scan, while the cap was not inserted during the VLF recordings; *ii*) the SNR is clearly lower in LF images than in HF images; *iii*) in the VLF images, although it is not difficult to recognize the phantom shape, the upper and bottom parts are blurred due to the fading of the sensitivity profile of the RF coils along the vertical direction. Despite these differences, we show that the images of the phantom are aligned by our co-registration pipeline, using the 9-parameter transformation (rigid + scaling).

In this procedure, we did not use the sliding-window approach since the image resolution was the same for the LF and HF datasets.

### 3.2 Brain data recorded at 46 μT and 1.5 T

We here describe features of ULF-MR brain images that make the co-registration with HF MRI nontrivial. First, the resolution of the ULF images is much poorer than that of HF images. While the HF images are recorded with a cubic voxel with 1-mm side, the voxels in the ULF MRIs are “rectangular cuboids” with size along the axial direction (6 mm) being twice the in-plane size (3 mm). These voxels are considerably larger than in HF images in order to increase the SNR and to reduce the recording time. In addition, the ULF dataset of the human brain acquired at LANL, as for the phantom dataset, is characterized by a resolution and contrast considerably lower than for the corresponding HF dataset. Finally, due to the smoothing effects of the large voxel size of the ULF images, a considerable blurring reduces the contrast between brain tissues, smoothing edges between different anatomical compartments and at the head edges. Indeed, edges detected by ULF are never sharp, in contrast with the HF images, making the detection of anatomical features and the co-registration between the two image sets harder.

The spectral content of both ULF and the re-sampled HF images was evaluated by means of the 2D power spectrum density (2D PSD). In S1 Fig, we show the 2D PSD for two slices of the dataset, calculated with ImageJ [38] using no-padding and a triangular filter window. The spectra of the HF images were calculated following the grouping procedure to reduce the resolution of HF images down to the ULF ones. The spatial frequency content of the two images is very different, with HF MRIs exhibiting higher frequencies (reflecting the edge sharpness between anatomical features) than ULF MRIs (due to the smoother edges).

**Fig 3:**
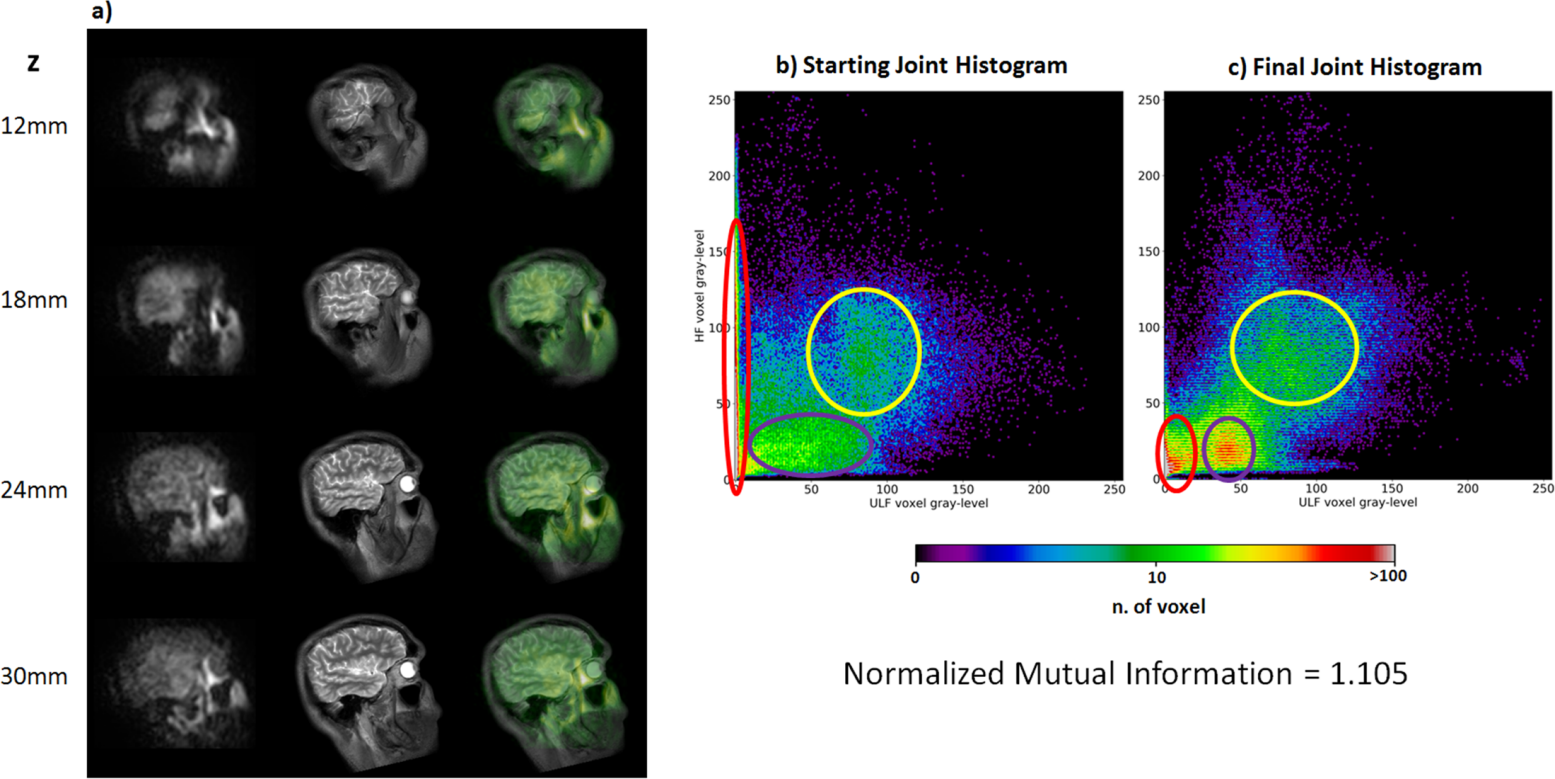
Co-registration of brain data recorded at 46 μT and 1.5 T. Panel a) Co-registration results on an *in-vivo* brain dataset recorded at 46 μT. a) Four sample slides. The left column represents the target ULF images, the middle column contains the HF co-registered images and the right column shows the overlap between the two image sets, where HF images are in grey tones and ULF images in green tones. b) and c) The joint histogram before and after co-registration, respectively. The red ellipses demarcate background voxels. These are badly aligned in the starting histogram, as suggested by the spread peak along the column corresponding to the background in the ULF image (0 gray value) and to background and head voxels in the HF image. In the final histogram the peak is concentrated close to the origin of the joint histogram, suggesting that background voxels are aligned in the final histogram. The violet ellipses represent some brain and skull structures that are misaligned in the starting configuration (the peak is wide); while in the final histogram a sharper peak is shown around ULF gray-level of 50. The yellow ellipses represent some structures with highest gray value like eyes and white matter that are less sharp in the starting joint histogram, while have a more clear structure in final configuration, demonstrating the good alignment of the images.

In Fig 3, the results from the SWIM co-registration of the *in-vivo* dataset are shown. In Fig 3A, we show a composite panel including three columns: the ULF dataset on the left side, the HF images after a rigid+scaling transformation in the centre, and the two overlapped datasets on the right, qualitatively suggesting that our approach was correctly co-registering the two sets of images. The value of the Normalized Mutual Information is 1.105.

In Fig 3B and 3C we show the joint histogram before and after co-registration, respectively. As explained in [18], if the images are correctly aligned and co-registered, the joint histogram is characterized by the presence of sharp peaks related to the aligned image features. Comparing the pre- and post-co-registration histograms, a visible modification of the histogram pattern can be appreciated. The red ellipses in Fig 3B and 3C mark a peak involving the small values of grey level in the ULF image, which are mainly associated with the background voxels. While before co-registration this peak spreads along the vertical axis (peak value = 1.23 e^3^, standard deviation on the *x* axis σ_*x*_ = 5, σ_*y*_ = 83), after co-registration it is restricted to small grey values for ULF and HF-MRI, suggesting a good overlap of background voxels (peak value = 7.25 e^4^, σ_*x*_ = 8, σ_*y*_ = 22). Peaks in Fig 3B and 3C highlighted by the violet ellipses are possibly related to part of the brain tissue and scalp represented by grey values ~40 in ULF images. These are misaligned in the histogram in Fig 3B, as indicated by the wide peak (peak value = 28, σ_*x*_ = 63, σ_*y*_ = 45); while a sharper peak (peak value = 87, σ_*x*_ = 32, σ_*y*_ = 23) in the histogram in Fig 3C is clearly visible, suggesting a good alignment. Finally, some structures with high grey value (eyes, white matter) are surrounded by the yellow contour. Again, the histogram before co-registration shows a smaller peak (peak value = 17, σ_*x*_ = 75, σ_*y*_ = 43) while the final histogram has a clearer pattern (peak value = 27, σ_*x*_ = 39, σ_*y*_ = 40), which demonstrates an alignment of these head structures. Note that the peaks representing the head structures do not lay on the bisection line, since the voxels within these structures are described by different distributions of grey values, which may depend on the different contrast at the two MRI fields.

**Fig 4:**
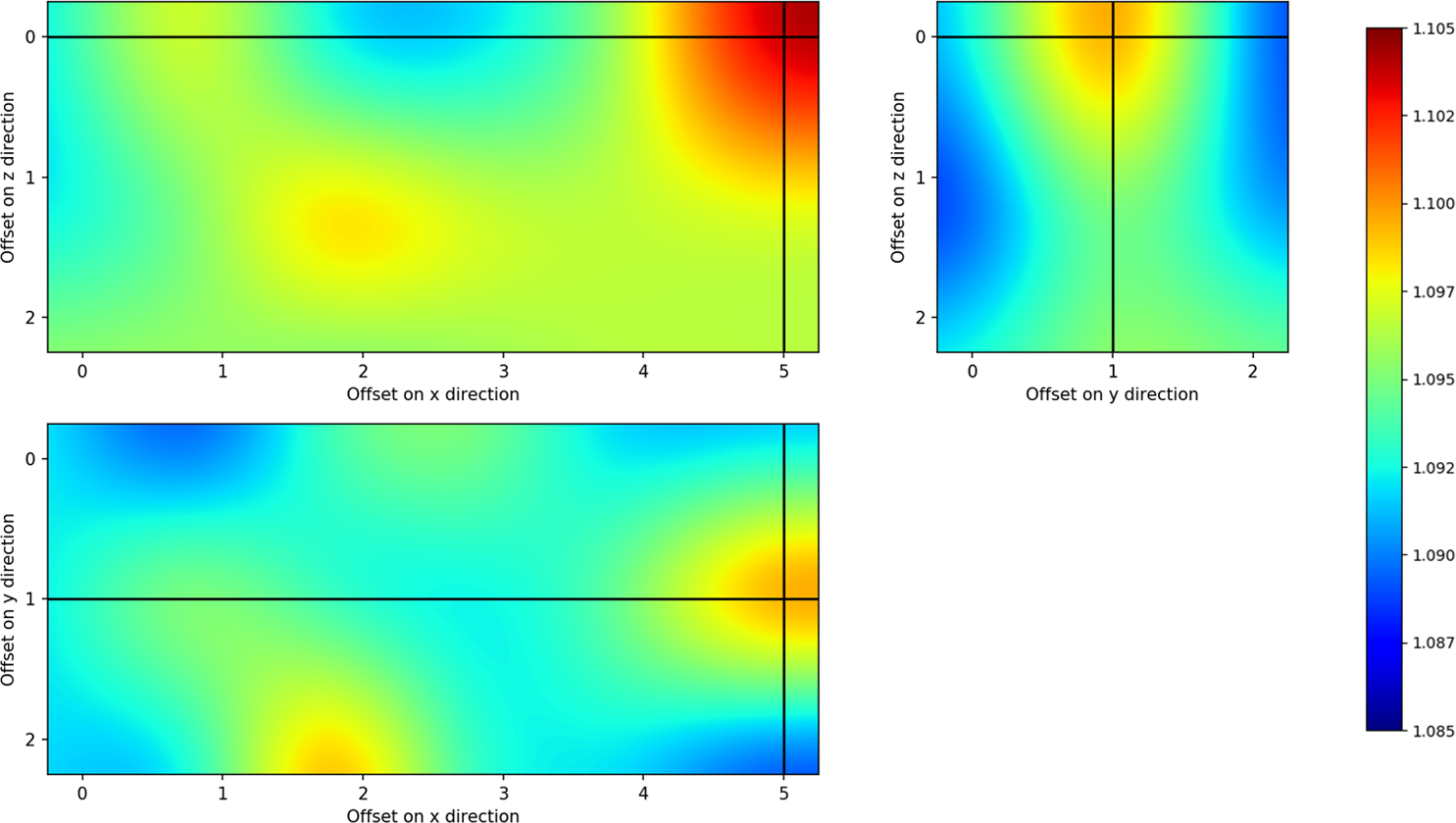
NMI as a function of different positions of the grouping window. The figure shows the interpolated NMI obtained by SWIM sliding the grouping window over the three direction of the *xyz* space. a) NMI for different offsets in *x* and *z* direction at *y*_off_ = 1; b) NMI for offsets in *y* and *z* directions at *x*_off_ = 5; panel c) shows the NMI for different offsets in *x* and *y* direction at x_off_ = 1. The cross-hair shows the highest value of NMI obtained at *x*_off_ = 5, *y*_off_ = 1, *z*_off_ = 0.

In Fig 4, we show the interpolated NMI as a function of the sliding window offsets in each direction at a fixed offset. According to formula (6), 6 offset values are possible along the *x* direction and 3 along the *y* and *z* directions. NMI changes with the offset values, suggesting that SWIM optimized the co-registration thanks to the sliding window approach. The cross-hair marks the best offset found by SWIM, corresponding to a window with an initial position with offsets equal to *x*_*off*_ = 5, *y*_*off*_ = 1 and *z*_*off*_ = 0.

**Fig 5:**
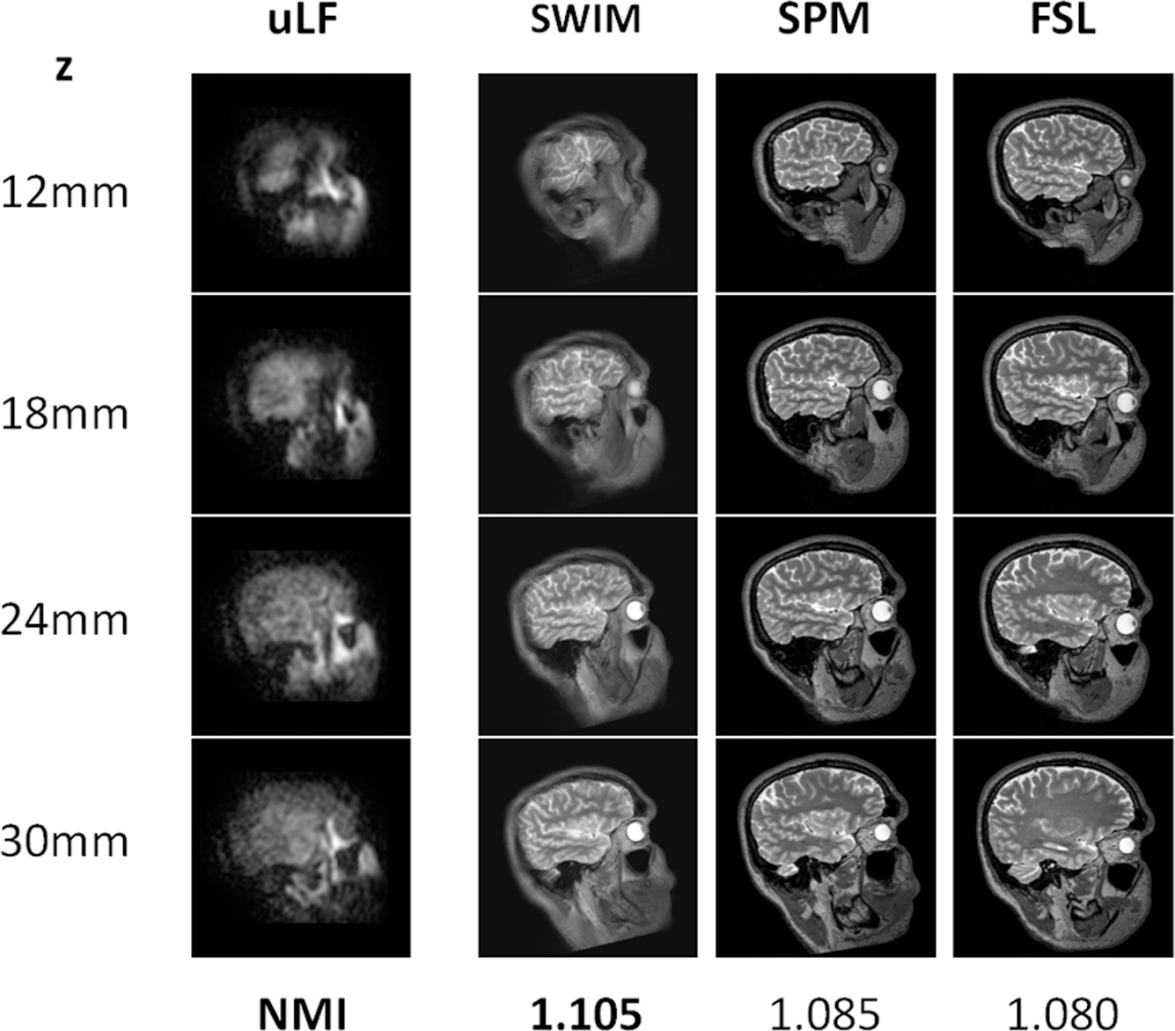
Comparison between SWIM and fMRI software packages. The results obtained with SWIM on the brain images recorded at 46 μT are compared with the outcomes of two different co-registration software packages routinely used for fMRI analysis. The corresponding NMI coefficients obtained after optimization are reported in the last row. The best result is obtained with SWIM. The co-registration procedure of fMRI processing software is very fast and efficient for fMRI analysis, but it is not adequate for co-registering ULF and HF images.

In Fig 5, the goodness of the co-registration obtained with SWIM is compared with the results obtained by FSL and SPM. In the first column, the target ULF images at four different *x* depths are shown, while the outputs of SWIM, and of FSL and SPM, applied on the HF images are shown in the other columns. Notably, the different software packages produce different alignments between the HF and ULF images, as suggested by the NMI values indicating the goodness of co-registration. We obtained the highest value of NMI by using our pipeline, demonstrating that the new procedure reaches the best performance in the superposition of HF-MRI images with the corresponding low resolution, noisy ULF-MRI images of the brain.

**Fig 6:**
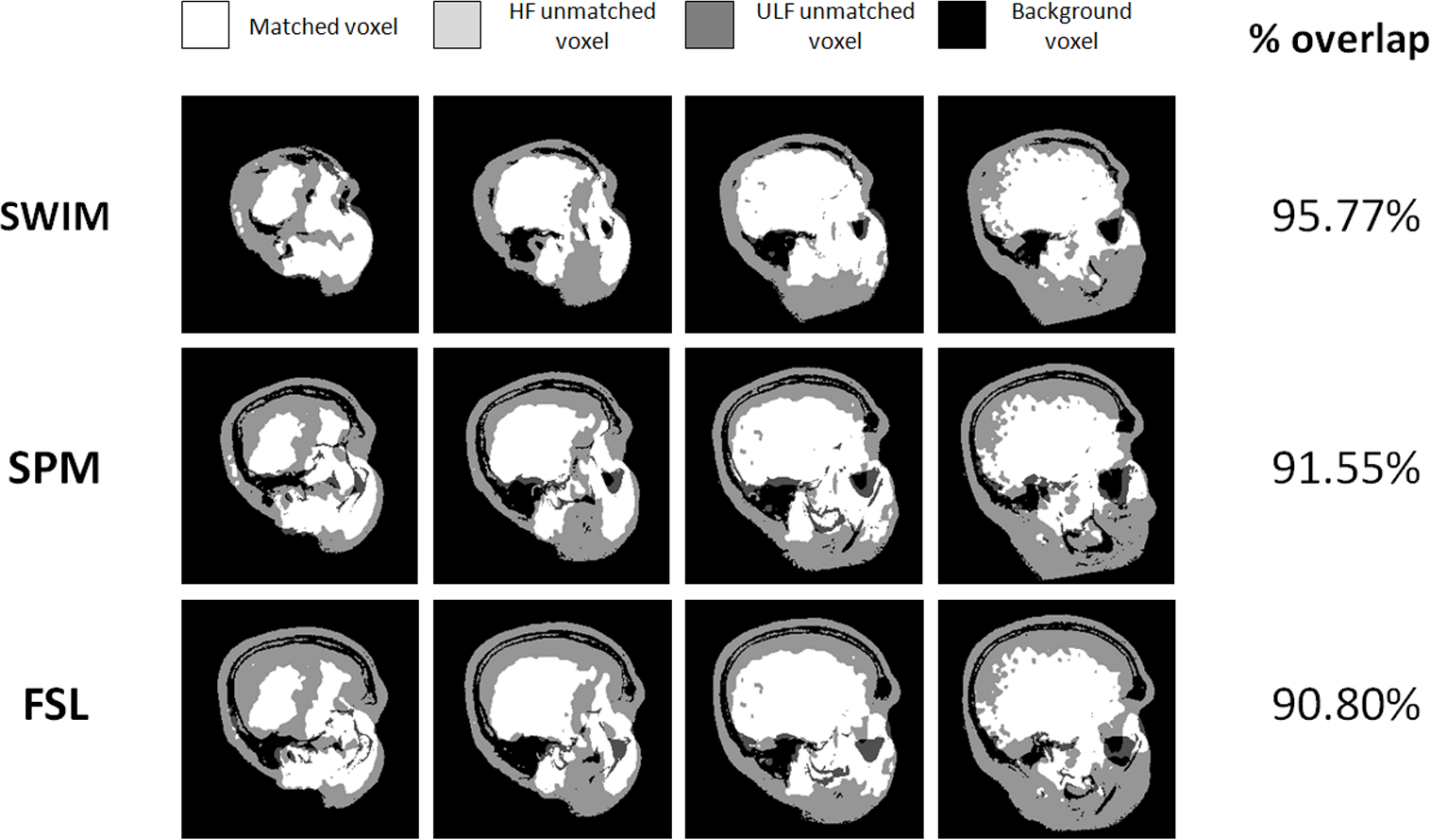
Overlap between the co-registered images. Segmented images are converted into binary format and the percentage of voxel overlap is reported on the last column for the three different co-registration procedures. Overlapping voxels are shown in white, light gray voxels are the HF voxels not matched in ULF image, dark gray indicates the ULF voxels not present in the brain shown in the HF scans and black indicates matching of the background. The matching of anatomical features is maximal for SWIM.

This indication is strengthened by analyzing the overlaps of the ULF and HF structures as obtained with the three methods, which are shown in Fig 6. The images were binarized after co-registration using an adaptive threshold filter [28,34] based on the grey level histograms of the two image sets. Fig 6 shows that using SWIM, anatomical features such as nasal sinuses and the cortical surface are well aligned. Conversely, using FSL and SPM, despite the good NMI value and plausible brain orientation, we did not obtain a good match of these structures (second and third row of Fig 6). The percentage of overlapping voxels is calculated considering only those voxels contained inside the HF head volume, while the external background voxels are not considered. Also ULF bright outliers outside the head surface marked by the HF structure were excluded. The final superposition found by our pipeline is characterized by the highest percentage of overlapping voxels.

Moreover, we report in Table 2 additional indices of either similarity (the higher the value, the higher the similarity–Kendall tau, Jaccard correlation, *R*^*2*^ score in addition to NMI) or dissimilarity (the lower the value, the higher the similarity—Correlation distance, Bray–Curtis distance, Mean squared error) estimated according to formulas shown in Table 1. Both similarity and dissimilarity indexes confirm the goodness of the alignment using our approach, which outperforms the co-registration results of the fMRI software packages.

**Table 2:** Similarity/dissimilarity indices for co-registration of HF and ULF MRI at 46 μT. Different values obtained for the indices in Table 1 applied to images co-registered using SWIM, FSL and SPM are reported in the table. The best values are highlighted in bold.

	SWIM	SPM	FSL
***Jaccard similarity***	**0.36**	0.34	0.32
***R***^***2***^ ***index***	**0.46**	0.28	0.20
***Kendall similarity***	**0.65**	0.62	0.55
***Bray-Curtis***	**0.30**	0.38	0.42
***Mean Squared Error***	**793**	1671	1978
***Correlation distance***	**0.26**	0.40	0.44

### 3.3 ULF brain data recorded at 50 μT

**Fig 7:**
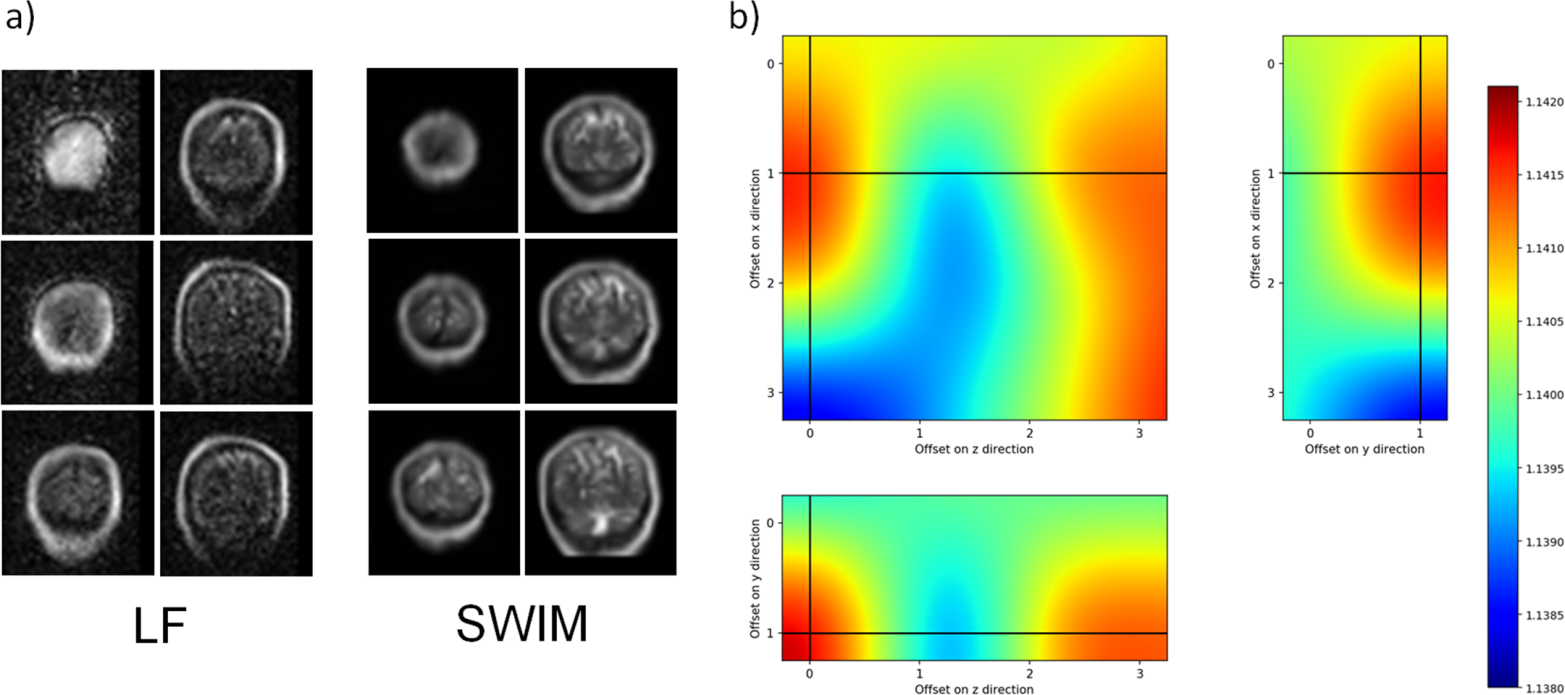
Co-registration of HF and ULF images at 50 μT. a) Co-registration of the dataset acquired at Aalto University. On the left the ULF image of the brain and on the right the down-sampled co-registered image at HF. b) NMI as function of the sliding window offset on the three (*x*, *y*, *z*) directions for *x*_off_ = 1, *y*_off_ = 1, *z*_off_ = 0.

Finally, we evaluated our pipeline using a dataset of the brain acquired with the system developed at Aalto University. This device records data from the occipital part of the brain. These LF images are blurred at the bottom and top due to the limited field of view. Fig 7A shows the coronal view of the ULF- and HF-aligned brain images, obtained with the *rigid+scaling* transformation. In Fig 7B, we show the interpolated NMI as a function of the sliding window offset in each direction at a fixed offset. According to formula (6), 4 offset values are possible along the *x* and *z* directions and 2 along the *y* direction. The intersection of plot lines marks the best offset found by SWIM, corresponding to a window with an initial position with offsets equal to *x*_*off*_ = 1, *y*_*off*_ = 1 and *z*_*off*_ = 0. The similarity/dissimilarity indices for this transformation are: Jaccard similarity = 0.393, *R*^*2*^ score = 0.354, Kendall’s similarity = 0.654, Bray–Curtis dissimilarity = 0.370, MSE = 1066, correlation distance = 0.293. In summary, also in the case of the images obtained from the Aalto system, SWIM was successful in the alignment, providing similarity/dissimilarity indices comparable with those of the Los Alamos system data.

### 3.4 Control analysis using a shear transformation

We further evaluated the performance of the SWIM approach, using only a *rigid* transformation, a rigid+scaling transformation, as well as using a shear transformation in addition to the rigid and scaling transformations described above. We always obtained an increase of NMI by including a scaling transformation, compared to a rigid transformation only, for all the datasets (~1.5% for the phantom, 0.15% for brain images). In contrast, we did not obtain better values of NMI by including a shear transformation, neither by tuning all parameters in a single run (~ –0.1% for the phantom, ~ –0.05% for brain images), nor by tuning separately the 3 shear parameters (~0.05% for the phantom, <0.01% for brain images). These results suggest that, at least for the analyzed datasets, the rigid+scaling transformation already provides a reliable co-registration.

### 3.5 Statistical assessment of SWIM

There is no ground truth to evaluate the accuracy of the registration of human brain images [39]. Therefore we applied two different approaches to assess the significance of our results: we estimated *i*) the robustness of the co-registration results using a consistency test and *ii*) the significance of our method using a permutation test.

The robustness of co-registration results was analyzed using the consistency test developed in Jenkinson et. al [39]. Specifically, a hundred randomly generated transformation matrices were used to modify the starting co-registration of the HF dataset to the ULF-MRI (first guess), without distorting the image structure. Then the coregistration pipeline was applied using these new images and the reference ULF images recorded at 46 μT.

The RMS deviation error is calculated between the transformation obtained with the generated images and the original one, using the following formula:

$$d_{RMS}=\sqrt{\frac{1}{5}R^{2}Tr\left(M^{T}M\right)+t^{T}t}$$ (8)

Where d_RMS_ is the deviation RMS measured, R is the radius of the ULF voxel and M and t are obtained from $\left(\begin{array}{cc} M & t\\ 0 & 0 \end{array}\right)=T_{j}∙A_{j}∙T_{0}^{-1}$, where T_j_ is the initial random transformation matrix, A_j_ is the matrix obtained after running the coregistration algorithm and T_0_ is the transformation obtained using the original HF dataset.

**Fig 8:**
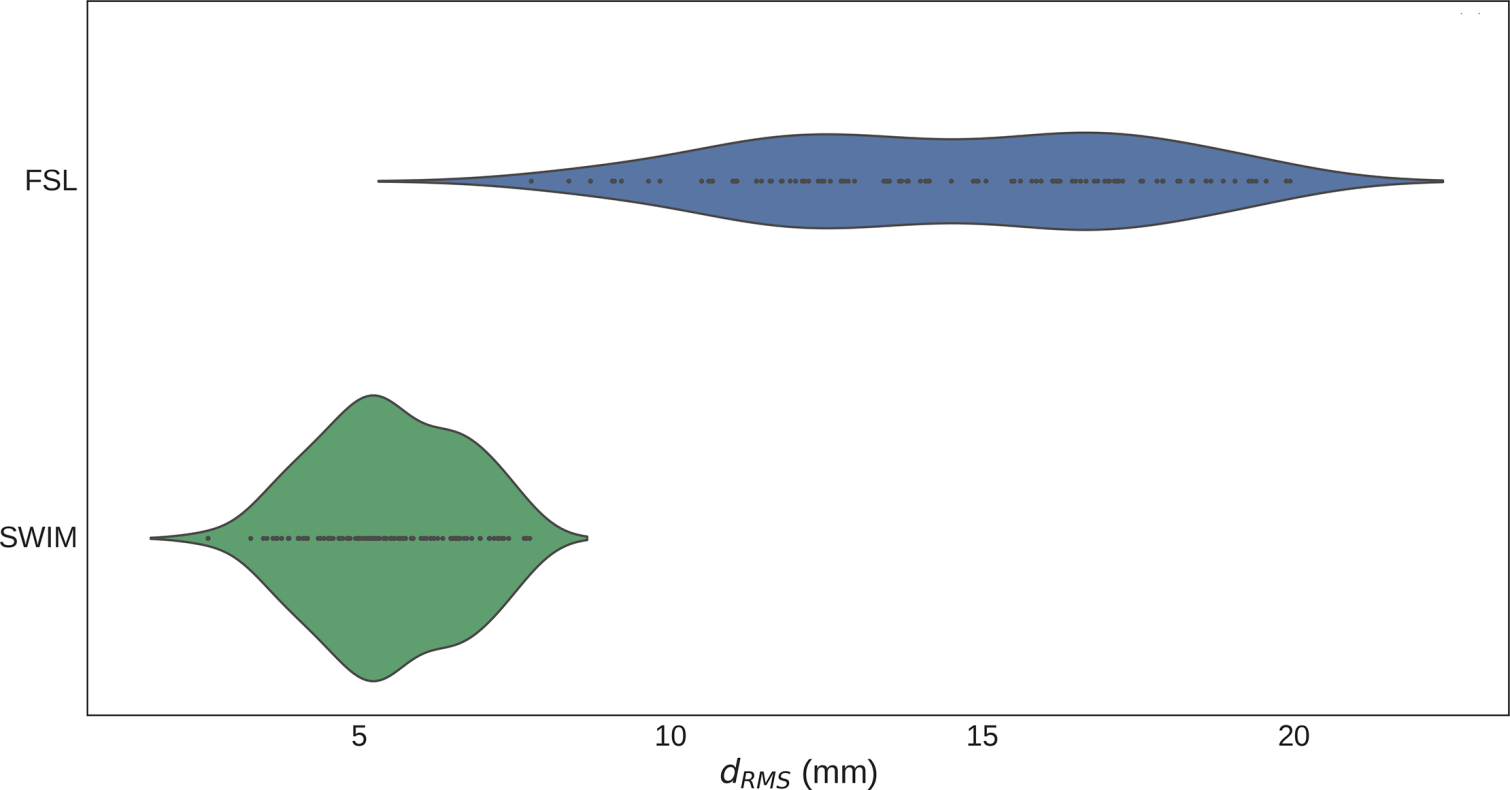
Distribution of d_RMS_ for SWIM and FSL. The violin plot of the distribution of d_RMS_ after running the consistency test over one hundred of different starting images for both SWIM and FSL, suggests that SWIM error is significantly lower than FSL coregistration error.

We calculate this index using both SWIM and FSL in order to get a distribution of errors. The mode of the deviation error for SWIM was about the size of the largest voxel dimensions (6mm) in the low resolution image, while for FSL two modes are found at about 2 and 3 times this size, respectively. The error of SWIM was significantly lower than that of FSL (p<0.001; unpaired t-test).The error distributions of SWIM and FSL are plotted in Fig 8.

We then used permutation test [40] to further evaluate if the transformation generated using the original dataset is obtained by chance. We shuffled voxels position of the HF image, destroying the structural information contained in the image but maintaining the distribution of the gray values. One hundred (100) randomly shuffled images were generated, and these were coregistered with the original LF image by our pipeline, in order to build a null distribution of the NMI. This distribution was used to evaluate if our coregistration transformation was obtained by chance.

For the ULF images recorded at 46 μT, the null distribution had a mean of 1.029 with a SD of 0.001. SWIM produced an NMI 1.105 which is significantly above chance level (p<0.01).

## 4. Discussion and conclusions

Here, we demonstrated that our approach based on minimization of the reciprocal of NMI supported by the sliding window grouping is robust to low SNR and contrast and is suitable to co-register ULF- and HF-MR images from phantom and *in-vivo* recordings obtained by different systems. The matching between image sets was quantified through a set of similarity indices in addition to NMI, proving that our approach provided transformation parameters which were more reliable than the ones obtained by other software packages used in the neuroscience community. Notably, we also demonstrated that 9 parameters (rigid transformation plus scaling) are adequate to achieve a reliable co-registration.

### 4.1 Methodological considerations

SWIM was designed to be robust to images with different SNR and contrast levels and with very different resolution. In a setting with different voxel resolutions in *x*, *y*, *z* directions, it is mandatory to scale the high-resolution images. This process leads to an information loss, which increases when the resolution difference becomes higher [28]. We used the sliding window to avoid bias due to unsuitable resampling of high-resolution images that could compromise image registration in the case of lower-resolution images with lesser SNR and contrast. As an example, co-registration results obtained by FSL were suboptimal, despite the relatively high NMI, probably due to a down-sampling strategy not adequate for this type of images. To speed up processing time, the initial coarse registration step in FSL is realized by sub-sampling the original images at a resolution of 8×8×8 mm^3^. In this way, the software quickly calculates a first guess before starting the co-registration using the original spatial resolution [41]. This method allows faster registration at a finer scale as a result of a reasonable initial estimate. Although the FSL procedure is very fast and efficient in fMRI analysis, in this particular case of ULF and HF brain images, the initial sub-sampling could cause a loss of information in the ULF structures, implying the calculation of a bad first guess for the subsequent image co-registration.

Moreover, the fact that the FOV in ULF-MR images represents only a part of the volume imaged by HF MRI could negatively affect the co-registration process since it limits the information contained in the dataset. In addition, the cost function (the reciprocal of NMI) includes a large number of local minima also due to the lower SNR of ULF-MRI images, and this number increases with the number of parameters used in the co-registration procedure. Overall, these effects generate a complex minimization hypersurface full of local minima where a minimization algorithm could be trapped [18,31]. The ASA algorithm, together with the sliding window down-sampling, is less sensitive to local minima, although the processing time is longer than in other minimization algorithms.

Our results suggest that, for the analyzed image sets, possible distortions are accounted for by different scaling parameters in the three dimensions of the HF and ULF grids, and only marginally by shear parameters, as suggested by the negligible increase of NMI when these are included. Future studies could test whether the use of regularization techniques may further improve the co-registration of this kind of datasets [42].

We designed SWIM to ensure portability across different VLF-HF and ULF-HF MRI scenarios. In this perspective, the pipeline could be improved using ad-hoc cost functions or minimization algorithms, leveraging on particular features of the imaging instruments. However, using a customized configuration would prevent from generalizing the pipeline to other MRI systems.

Moreover, SWIM could be effectively adapted to images from different modalities to track changes in tissues or in material structures in order to detect the effect of therapeutic treatments or micro-morphology changes [30,43]. Since the implementation of reliable and robust methods of multimodal image co-registration and image fusion [44–46] is central in several medicine-related research and clinical fields [42], it would be worth exploiting our pipeline in other imaging modalities in the future.

### 4.2 Impact of optimized co-registration on LF-MRI

The application of optimized co-registration software would support the exploitation of LF MRI for the following reasons. Co-registering ULF MRI to HF MRI is of fundamental importance for instruments integrating MRI with MEG in the same setup, with the aim at improving the spatial accuracy of MEG. Source localization requires the solution of an ill-posed inverse problem [47], for which, in addition to source models, head (volume conductor) models are used to restrict the possible solutions, and to associate magnetic field sources to specific brain areas. These models are usually obtained from the HF MRI of the subject’s head, which should be co-registered in the same reference system as the MEG.

This is achieved through two co-registrations, one between the fiducial markers and the anatomical image of the subject’s head and the other between the reference systems of the subject’s head and the MEG sensor space. Thus, co-registration of HF-MRI to MEG [48,49] has an influence on MEG spatial accuracy and reliability. Measuring both MEG and ULF MRI with the same sensor setup implies, assuming that proper calibrations have been performed, that anatomical and functional images are already co-registered, together with the position of the subject’s head in the MEG sensor space (thanks to the image of the head produced by ULF-MRI) [7,12,14]. On the other hand, for practical usage of present day ULF-MRI and MEG systems, due to the low resolution of ULF-MRI images, a co-registration of HF-MRI to ULF-MRI is needed. This co-registration relies on a larger number of points than the one using fiducial markers, and it can therefore be expected to be far more reliable and accurate. In this work, we have presented a new co-registration method and we have compared it to other existing methods. We found that our method has greater robustness. The impact of this improved co-registration on MEG localization is under investigation and will be the subject of another work.

In addition, ULF MRI could provide information on electrical conductivity of different head compartments; this information could be used to further improve localization accuracy [50]. However, current ULF-MRI systems compatible with MEG allow to image only a part of the brain to keep the measurement duration reasonable; also, the contrast and resolution in these images are still inadequate to build an accurate volume-conductor model. Therefore, data analysis would still require an independently acquired high-resolution MRI. Our approach was able to successfully co-register phantom and *in-vivo* LF images obtained with different systems to the respective HF images. The obtained co-registration was better than the one obtained with fMRI analysis packages, as assessed through multiple similarity/dissimilarity indices.

The development of a new generation of NMR and MRI apparatuses working at ultra-low measurement fields is opening up new possibilities to image and characterize properties of biological structures. ULF MRI can be used to detect different brain tissues, usually not distinguishable at HF strengths, using particular sequences and leveraging on different tissue relaxation times, with the possibility to discriminate between different brain tumors without contrast agents [51]. ULF MRI has already revealed abnormal relaxation rates in prostate cancer tissues, which correlated with the percentage of prostate tumor tissue [11]. For practical scientific and clinical applications on living tissues, instruments capable of providing images with very high quality and good spatial resolution in a reasonable recording time are needed. The present-day ULF MRI systems are still at an early stage although their output could in principle be used in a multimodal approach together with HF MRI and/or other diagnostic techniques such as X-ray computed tomography, ultrasonography and biopsy. In this perspective, our approach could enhance exploitation of multimodal imaging involving VLF and ULF MRI.

## Supporting information

S1 Fig Power spectrum density of HF and ULF MRI at 46 μT. a) Spectrum of the ULF image. The spectrum is mainly characterized by low-frequency components, indicating low contrast and blurring. b) Spectrum of the HF image. The spectrum includes also high frequencies and indeed the edges between different anatomical regions are clearly detectable. Notably, the HF image was down-sampled to the same spatial resolution as the ULF image.
(PNG)
