# Supplementary figures and images for: Optimized 3D co-registration of ultra-low-field and high-field magnetic resonance images
Source: PLoS One. 2018 Mar 6;13(3):e0193890. doi: 10.1371/journal.pone.0193890 (PMC5839578; doi:10.1371/journal.pone.0193890)

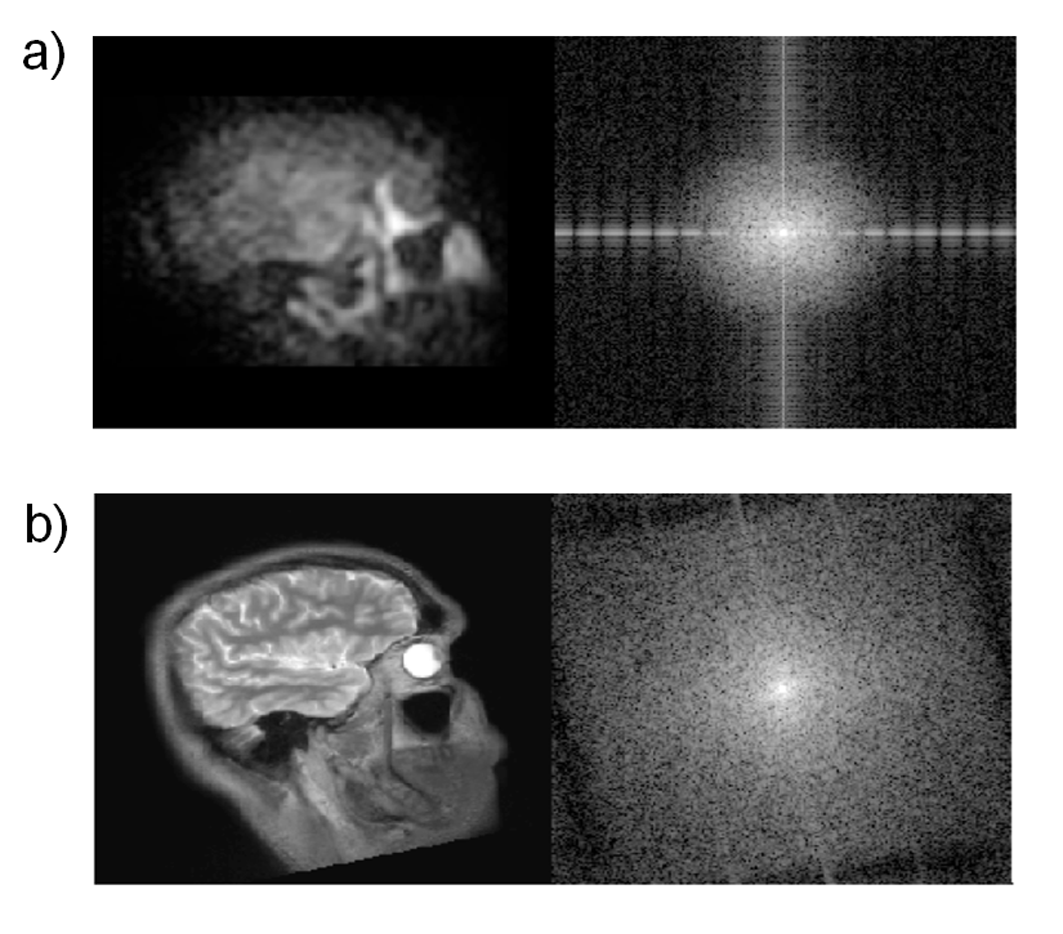

Supplement: S1 Fig — a) Spectrum of the ULF image. The spectrum is mainly characterized by low-frequency components, indicating low contrast and blurring. b) Spectrum of the HF image. The spectrum includes also high frequencies and indeed the edges between different anatomical regions are clearly detectable. Notably, the HF image was down-sampled to the same spatial resolution as the ULF image. (PNG) [file pone.0193890.s001.png]
